# Global Consequences of Liver Ischemia/Reperfusion Injury

**DOI:** 10.1155/2014/906965

**Published:** 2014-04-01

**Authors:** Constantinos Nastos, Konstantinos Kalimeris, Nikolaos Papoutsidakis, Marios-Konstantinos Tasoulis, Panagis M. Lykoudis, Kassiani Theodoraki, Despoina Nastou, Vassilios Smyrniotis, Nikolaos Arkadopoulos

**Affiliations:** ^1^Second Department of Surgery, School of Medicine, Aretaieion University Hospital, University of Athens, 76 Vassilisis Sofia's Avenue, 11528 Athens, Greece; ^2^Second Department of Anesthesiology, School of Medicine, Attikon University Hospital, University of Athens, 1 Rimini Street, 12462 Athens, Greece; ^3^Division of Surgery & Interventional Sciences, Royal Free Hospital Campus, University College London, 8 South Pond Street, Hampstead, London NW3 2QG, UK; ^4^First Department of Anesthesiology, Aretaieion Hospital, University of Athens School of Medicine, Vassilissis Sofias 76, 11528 Athens, Greece; ^5^Fourth Department of Surgery, School of Medicine, Attikon University Hospital, University of Athens, 1 Rimini Street, 12462 Athens, Greece

## Abstract

Liver ischemia/reperfusion injury has been extensively studied during the last decades and has been implicated in the pathophysiology of many clinical entities following hepatic surgery and transplantation. Apart from its pivotal role in the pathogenesis of the organ's post reperfusion injury, it has also been proposed as an underlying mechanism responsible for the dysfunction and injury of other organs as well. It seems that liver ischemia and reperfusion represent an event with “global” consequences that influence the function of many remote organs including the lung, kidney, intestine, pancreas, adrenals, and myocardium among others. The molecular and clinical manifestation of these remote organs injury may lead to the multiple organ dysfunction syndrome, frequently encountered in these patients. Remote organ injury seems to be in part the result of the oxidative burst and the inflammatory response following reperfusion. The present paper aims to review the existing literature regarding the proposed mechanisms of remote organ injury after liver ischemia and reperfusion.

## 1. Introduction

Liver ischemia and reperfusion has been the topic of intense study during the last decades since it is implicated in many clinical scenarios, including hemorrhagic shock and resuscitation, trauma [1], liver resections [2], and liver transplantation [3].

After the introduction of vascular control techniques during hepatic surgery, liver ischemia and reperfusion has been recognized as one of the key elements that contribute to postoperative morbidity and mortality [4]. Liver dysfunction and failure are serious postoperative complications which may ensue as a result of reperfusion injury. Although the liver has been studied in the aforementioned clinical scenario (i.e., hepatic surgery) and liver dysfunction is widely recognized as a consequence of hepatic reperfusion injury, many other remote organs seem to be influenced during this process as well [5–7].

The aim of this paper is to review the existing literature and summarize the proposed mechanisms of remote organ injury that are mediated by liver ischemia/reperfusion injury in hepatic surgery.

## 2. Liver Injury

The topic of liver reperfusion injury has been extensively investigated in the context of hepatic surgery and therefore it is not the aim of the present review to describe in detail the pathophysiology of this entity.

Briefly, liver ischemia/reperfusion injury can be divided into two distinct phases. Ischemia seems to be the initial insult to the organ, which, although tolerable from the liver, triggers the production of molecules that are essential for the induction of reperfusion injury. During this phase intracellular production of xanthine oxidase and NADPH oxidase from liver cells is promoted [3]. The early phase of reperfusion occurs from the first minutes after ischemia and up to 6 hours later. Immediately after reperfusion, cellular swelling takes place due to the disturbance of NA/K/ATPase function [8]. In addition, reactive oxidant species (ROS) can be found inducing oxidative stress to the liver as well as to distant organs [9, 10]. ROS activate Kuppfer cells, promoting even further ROS as well as cytokine production. In the meantime, NO levels are reduced and there is an imbalance between endothelin-1 and nitric oxide (NO) production from NO synthase (NOS), leading to vasoconstriction of the sinusoids [11]. The narrowing of the sinusoids leads to entrapment of platelets and neutrophils. Hepatocyte injury is promoted through hepatocellular necrosis and apoptosis, though distinct pathways [12–14].

An excessive inflammatory response is clearly recognized as a key mechanism of injury during reperfusion. Local production of cytokines (IL-1b and TNF-*α*) is the precursor of the late phase of reperfusion injury [3]. This is the cellular phase and is characterized by activation and migration of neutrophil, CD4+ T lymphocytes, and platelets into the liver. Cell-surface adhesion molecules (such as intracellular cell adhesion molecule, ICAM, and vascular cell adhesion molecule, VCAM) are expressed in the hepatocytes and endothelial cells and inflammatory cell adherence takes place [15]. These cells are trapped in the constricted and narrowed sinusoids and result in inflammatory injury and microvasculature failure. CD4+ T lymphocytes produce granulocyte-macrophage colony-stimulating factor, interferon gamma, and TNF-*β*, which amplify Kupffer cell activation and cytokine release [15].

Although intravascular coagulation during inflow occlusion can reduce or block blood flow in sinusoids and cause liver injury, active vasoconstriction and cell entrapment seems to be the major contributing factor to microcirculatory problems [15]. Microcirculatory failure leads to aggravated and prolonged ischemia, as parts of the liver remain hypoxic, aggravating necrosis, Kupffer cell activation, further cytokine and ROS release, and creating a vicious cycle of excessive inflammatory response, reactive oxygen and nitrogen species production, and further oxidative tissue injury [4, 15].

**Figure 1:**
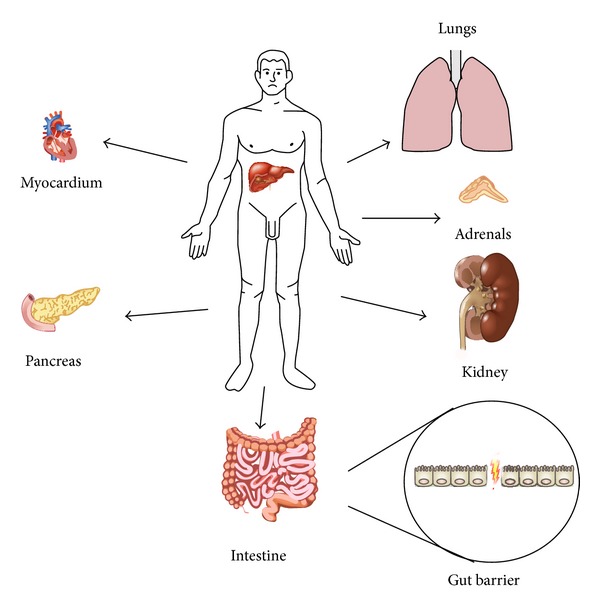
Distant organs that have been implicated in the remote injury after hepatic ischemia/reperfusion.

Although the many aspects of liver reperfusion injury remain to be elucidated, there is extensive literature on the mechanisms that mediate liver injury. However, only recently has there been research concerning the mechanisms involved in remote organ injury after the initial insult to the liver. Distant organs that have been implicated in the remote injury after hepatic ischemia/reperfusion are depicted in Figure 1.

## 3. Kidney Injury

**Figure 2:**
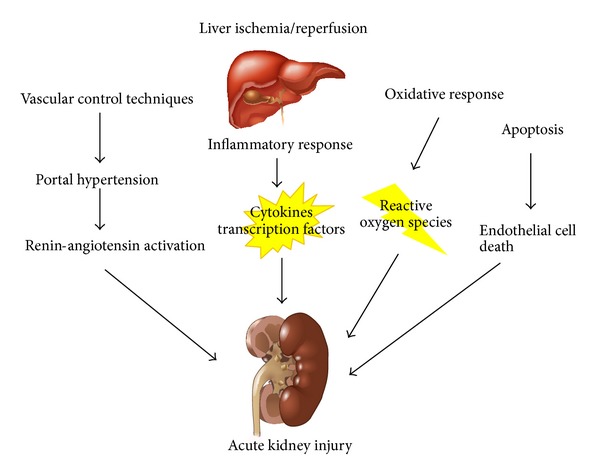
Mechanisms involved in remote injury to the kidney after liver ischemia/reperfusion.

Ischemia/reperfusion injury of the liver remnant or donor liver is a frequent cause of acute liver failure (ALF) during the perioperative period and is a common complication after major liver resection or liver transplantation [4, 16, 17]. Acute kidney injury (AKI) occurs frequently in patients with ALF and poses a serious clinical problem in the perioperative period [18, 19] increasing the associated morbidity and mortality. The reported incidence of AKI ranges from 40 to 85% [18, 20] and in the setting of liver transplantation can be as high as 95% [21]. Unfortunately, the pathophysiology of AKI associated with liver ischemia/reperfusion injury is not fully understood. However, there is emerging evidence suggesting multiple molecular mechanisms. The basic mechanisms are summarized in Figure 2.

The initiating event appears to be portal hypertension, which is the result of portal vein occlusion, incorporated in various techniques of vascular control of the liver used during hepatic surgery. This induces splanchnic vasodilation with subsequent intrarenal vasoconstriction [20, 22, 23]. Splanchnic vasodilation leads to hypotension [20] which in turn leads to activation of the renin-angiotensin system [20, 24]. Upregulation of renin-angiotensin system can cause severe reduction of glomerular filtration rate, urinary sodium excretion, and free water excretion. It has been proposed that intense intrarenal ischemia subsequent to renin-angiotensin activation leads to renal tubular necrosis and renal dysfunction.

However, other mechanisms may play an important role in the pathogenesis of renal dysfunction after liver ischemia/reperfusion injury. Systemic inflammatory response may induce renal injury [20, 25]. Circulating levels of proinflammatory cytokines and transcription factors, including interleukin (IL)-6, tumor necrosis factor-alpha (TNF-*α*), and high mobility group box (HMGB) 1, are increased and their release from the liver may promote inflammatory changes in the kidney after liver ischemia/reperfusion [25–28]. Kupffer cells activation plays the predominant role in the production and release of these cytokines. Recently, Park et al. reported the significant role of Paneth cell derived IL-17A in the aggravation of the observed systemic inflammatory syndrome and the exacerbation of renal injury [29]. These proinflammatory factors can upregulate endothelial adhesion molecules in distant organs including the kidney. Upregulation of renal endothelial adhesion molecules including E-selectin, P-selectin, and intercellular adhesion molecule (ICAM)-1 promotes leukocyte recruitment and extravasations to the renal interstitial space [30, 31]. Meyer et al. reported that liver ischemia/reperfusion results in significant ICAM-1 upregulation in remote tissues, including the kidney, and is likely mediated by cytokines such as TNF [32]. In addition, circulating bile acids and endotoxin and circulating immune complexes may contribute to the development of AKI [20].

The integrity of the endothelial barrier plays a pivotal role in the protection against AKI, by regulating leukocyte recruitment to the area of injury [25, 30]. Lee et al. reported that hepatic ischemia/reperfusion injury produces marked endothelial cell apoptosis in the kidney, which is more prominent in the interstitial capillary endothelial cells [33]. Endothelial cell death due to apoptosis would impair the regulation of leukocyte infiltration, further exacerbating renal injury. Apoptotic cell death represents the execution of an ATP-dependent process often initiated by death ligand/death receptor interactions, such as Fas ligand with Fas [34, 35].

Except from the aforementioned mechanisms, oxidative stress is also a major determinant of liver ischemia/reperfusion induced AKI. Activated neutrophils [33] aggregate in the subendothelial space, where they release reactive oxygen species (ROS), enzymes, and cytokines, causing direct renal injury [36] and the recruitment of monocytes and macrophages leading to further aggravation of the oxidative injury. Kadkhodaee et al. reported that liver ischemia/reperfusion causes renal injury including oxidative status changes as shown by the increase in renal malondyaldehyde (MDA) levels and the decrease in superoxide dismutase (SOD) and catalase activity [28]. Similar results were reported in other studies showing that hepatic ischemia and reperfusion may cause oxidative stress to renal tissue and that administration of antioxidants seems to have a beneficial effect via the glutathione (GSH) system and the modulation of MDA levels [37, 38]. The pivotal role of reactive oxygen species in the development of AKI was also demonstrated by the beneficial effect of hepatic ischemic preconditioning and the administration of free radical scavengers [39]. However, although high concentrations of oxygen free radicals induce tissue injury during the reperfusion period after prolonged ischemia, moderate oxygen free radicals are an important prerequisite of ischemic preconditioning in renal cells [40]. Oxygen free radicals phosphorylate several important cytoprotective kinases, including extracellular signal-regulated kinase (ERK1/2), mitogen activated protein kinase (MAPK), and Akt, and are involved in the upregulation of several cytoprotective genes [41]. ROS are attractive signaling candidates to account for preconditioning in the kidney because renal cells are subject to obligatory bursts of oxidative stress during the reperfusion phase after each preconditioning stimulus. Therefore, distant renal exposure through the blood stream of moderate ROS caused by hepatic ischemic preconditioning may also initiate cytoprotective signaling to defend against subsequent and more severe free radical-mediated injury in renal tubule cells.

Damage of the actin cytoskeleton, which contributes to the development of AKI, is another proposed mechanism [42, 43]. Lee et al. have shown that liver ischemia/reperfusion promotes renal F-actin breakdown [33]. Additionally, F-actin disruption induces apoptosis in several cell lines [44]. Therefore, loss of actin cytoskeleton may contribute to the development of renal tubular and endothelial apoptosis.

At a molecular level, adenosine has been proposed to play a pivotal role. Adenosine release is increased after stress (e.g., hypoxia, ischemia/reperfusion) with subsequent activation of adenosine receptors (ARs), which protect against cell death in several cell types [45]. Endogenous adenosine production is crucial in protecting against ischemia-induced organ injury. Activation of cell surface Adenosine 1 (A1) ARs produces cytoprotective effects in many organ systems including the kidney [46]. A1AR activation produces several cellular effects that are suited to attenuate the multifaceted pathophysiology of AKI (endothelial and renal tubular cell apoptosis, inflammation, and necrosis). Acute renal protection after hepatic ischemia/reperfusion with A1AR activation is mediated by Akt activation [47]. In particular, Akt has diverse functions to counteract apoptosis including inhibition of mitochondrial cytochrome c and phosphorylation of several proapoptotic factors (e.g., caspase 9, glycogen synthase kinase 3) [48]. Akt can also increase the activity of heat shock protein 27 in certain cell types [49, 50] promoting F-actin stability. Better preserved F-actin cytoskeleton in the kidneys may contribute to reduced renal tubular necrosis and apoptosis [33].

Another suggested molecular pathway is the protease-activated receptor 1. As reported by Park et al. modulation of the receptor through the administration of activated protein C resulted in reduced expression of several proinflammatory genes, reduced kidney filamentous-actin degradation and neutrophil infiltration, and better preservation of vascular permeability of the kidney after liver ischemia/reperfusion [51]. Except from this receptor, the family of Sphingosine-1-phosphate (S1P) receptors have been shown to play a role either in the protection or the development of ischemia/reperfusion induced AKI. More specifically, S1P1 receptor activation via S1P provides a protective signaling cascade, whereas S1P3 activation can potentially initiate detrimental effects. The authors also showed that the protective effect of S1P activated S1P1 receptor was induced via pertussis toxin-sensitive G protein (Gi/o) ERK and Akt-mediated pathways [52] and included reduced necrosis, inflammation and apoptosis, enhanced preservation of F-actin cytoskeleton, improved vascular integrity, and reduced neutrophil infiltration [53].

Endogenous hydrogen sulphide has also been demonstrated to play a role as a modulator of key peroxidant and inflammatory events occurring after total hepatic ischemia and reperfusion, attenuating lipid peroxidation and inflammation reactions by reducing MDA, nuclear factor-*κ*B (NF-*κ*B), and ICAM-1 production [54]. Finally, Suzuki et al. have proposed a putative role for platelet activating factor (PAF) and endothelin (ET)-1 in the pathogenesis of renal injury after hepatic ischemia and reperfusion. Specifically, they reported that the pretreatment with a PAF receptor antagonist resulted in attenuation of renal injury [55].

## 4. Lung Injury

The impact of pulmonary complications on morbidity and mortality after hepatic ischemia/reperfusion was acknowledged more than 2 decades ago [56, 57]. It was soon realized that lung injury resulted from the reperfusion of the donor liver during transplantation, a condition that also ensues during extensive hepatectomies. One of the proposed mechanisms is the release of TNF-*α* from reperfused Kupffer cells, which interacts with pulmonary capillaries and elicits the expression of adhesion molecules, such as ICAM-1 and E-selectin, leading to migration of neutrophils and subsequent lung injury [58–60]. In combination with TNF-*α*, a variety of proinflammatory molecules, such as PAF, cytokine-induced neutrophil-chemoattractant protein [61], IL-6, and IL-18 [62, 63], as well as substance-P [64], are released from the reperfused liver and have been found to mediate lung injury after hepatic ischemia/reperfusion. On the contrary, IL-4 and IL-10 seem to exert a protective role [63, 65].

Cytokine production by the reperfused liver seems to provoke a sustained production of cytokines locally in the lung, such as TNF-*α* from alveolar macrophages, macrophage inflammatory protein (MIP)-2, and the pathway of interleukin-6/signal transducer and activator of transcription-3 (STAT3) [60, 65, 66]. The importance of the locally sustained pulmonary inflammation was underlined by the suppression of pulmonary NF-*κ*B through administration of IL-10, which was followed by cytokine suppression and prevention of hepatic ischemia/reperfusion-induced lung injury [65].

Another important mechanism is the translocation of endotoxin to the systemic circulation. Bacterial translocation is evident after liver resection under vascular control even after the creation of a portasystemic shunt [67]. In particular, insufficiency of Kupffer cells during reperfusion allows the spill-over of endotoxin in the pulmonary capillaries, stimulating TNF-*α*, IL-6, epithelial neutrophil activating protein-78, and MIP-2 production and subsequent neutrophil infiltration of the lungs [68, 69]. The protective action of inhibition of endotoxin against hepatic and pulmonary injury after hepatic ischemia/reperfusion alone or in combination with hepatectomy further strengthens the role of endotoxin in the pathophysiology of the syndrome [70].

Oxidative stress during hepatic ischemia/reperfusion has been consistently shown to play a crucial role in the development of lung injury. Although the lung resists oxidative stress by upregulating antioxidant enzymes, exogenous antioxidants offer significant benefits [7]. Xanthine oxidase (XO) has been shown to release hydroxyl and superoxide radicals during reperfusion. Its inhibition by allopurinol has been reported to ameliorate lung injury in this setting [71, 72]. Data from our laboratory have also marked nitrosylation of pulmonary proteins as a prominent feature in liver ischemia/reperfusion induced lung injury [60]. However, production of NO seems to protect the lung from injury [73–75]. In a recent work, we have also shown the role of oxidative reactions mediated by free iron in this setting, and the significant improvement of hepatic ischemia/reperfusion-induced lung injury by iron chelation [7]. Improvement has also been offered by other antioxidants, such as the general anesthetic propofol, methylene blue, and mannitol [76–78]. Recently, preconditioning of the lungs with isoflurane showed promising results, although the involved mechanisms remain poorly understood [79].

Lung injury and acute respiratory distress syndrome can severely complicate the postoperative course following liver transplantation [80]. Hepatic ischemia/reperfusion-induced lung injury may be implicated in these complications, due to the release of cytokines in the bloodstream, such as TNF-*α* and IL-6 and IL-8 [81, 82], as well as due to release of endotoxin, which seems to prolong ventilatory support [57, 83]. Whether this release of endotoxin and priming of neutrophils is responsible for the susceptibility of these patients to transfusion-related lung injury remains to be shown. Nevertheless, acute lung injury after liver transplantation has been repeatedly correlated to platelet transfusion [84–86].

## 5. Gut Injury

Gut barrier failure in the form of bacterial and/or endotoxin translocation has been reported following liver ischemia/reperfusion, either during hepatic resections with the use of the Pringle maneuver or after liver transplantation, both in the experimental and clinical setting [67, 87, 88]. However, liver ischemia/reperfusion injury has been implicated in other forms of intestinal dysfunction, including motility, transit time, and absorption function changes [89].

Multiple mechanisms have been proposed, but the exact pathophysiology remains to be elucidated. Experimental data suggest that intestinal mucosa oxidative injury results from the effect of liver-produced ROS, which are “spilled” in the systemic circulation. This is demonstrated in small animal model experimental studies. Okay et al. have reported decreased intestinal mucosal MDA concentration, attenuated intestinal mucosa injury, and bacterial translocation 24 hours after extensive hepatectomy in rats, following the administration of the antioxidant N-acetylcysteine [90]. Alexandris et al. have also reported decreased levels of protein carbonyls and decreased endotoxin translocation after antioxidant treatment, following extensive hepatectomy in rats [91]. The proposed mechanism of remote oxidative injury to the gut mucosa from liver derived ROS is damage of the tight junctions between enterocytes, which results in increased permeability and gut barrier failure.

Another suggested mechanism is congestion of the portal venous system. During the Pringle maneuver, blood from the intestine and pancreas is pooled in the portal venous system leading to portal hypertension. It has been demonstrated by Filos et al. that rats subjected to the Pringle maneuver for 30 minutes had immediate (30 minutes) and delayed (24 hours) gut barrier failure with increased bacterial and endotoxin translocation, which might be attributed to portal stasis leading to intestinal congestion. Bowel wall edema may lead to intra-abdominal hypertension resulting in further compromisation of intestinal perfusion [92] and gut barrier dysfunction [93]. In addition, sinusoidal microcirculatory failure during reperfusion decreases the total vascular bed of the liver, increasing intrahepatic portal resistance and leading to postoperative portal hypertension [94]. However, this scenario is only applicable in cases of liver ischemia/reperfusion injury following liver surgery, where vascular occlusion techniques are applied.

Postoperative liver failure seems to be one of the most serious complications after hepatic surgery, when liver ischemia and reperfusion occurs. Many of the factors which comprise gut barrier function seem to be compromised in postoperative liver failure [95]. Bile production and intestinal motility are decreased [89, 96, 97]. Bile has a trophic role for the intestinal mucosa. It binds to intraluminal endotoxin and bacteria creating nonabsorbable complexes and contains secretory IGA, which has antibacterial properties. The decreased intestinal motility results in increased intestinal bacterial load, which in turn has been shown to cause intestinal mucosal injury [97].

Apoptosis is implicated in gut mucosal injury following liver ischemia and reperfusion, since it is already known that extracellular free radicals can induce cell apoptosis [98–100]. It has been reported that oxidative stress can induce damage in enterocyte cell membranes and DNA, activating apoptotic pathways and thus disrupting the mucosal barrier [101, 102]. Ikeda et al. have shown that the typical intestinal oxidative injury after ischemia and reperfusion is the “lifting” of the epithelial layer from the lamina propria and that apoptosis and necrosis are the main involved mechanisms [103]. During this process, enterocytes create subepithelial crypts and “blubs”—microscopically known as Gruenhagen's space [104]. Alterations in the expression of adhesion molecules between the cellular membrane and the matrix in the villi may be responsible. Beaulieu has implicated changes in the expression of B1 integrins [105], while Probstmeier et al. have reported changes in the expression and function of the complex molecule J1/tenascin in enterocytes located at the tip of the villi [106]. It is possible that oxygen free radicals damage these molecules leading to loss of interaction between enterocytes and matrix. It has been shown that loss of this interaction can lead to apoptotic cell death [107].

Remote intestinal injury has been reported by many authors in the setting of liver transplantation and liver ischemia and reperfusion protocols involving hepatectomy either under vascular control or not. Mochida et al. suggested that bacterial translocation, following liver transplantation in rats, is responsible for hypercoagulopathy in the graft's sinusoids, reversing it with oral antibiotic therapy [108]. Lemaire et al. described a porcine model of severe, extraintestinal tissue injury, consisting of prolonged hepatic ischemia and reperfusion, in combination with hemihepatectomy. Venous congestion of the gut during ischemia was prevented with a temporary portal-caval shunt. This prevented direct congestive damage to the gut, associating injury with remote ischemia/reperfusion. They reported bacterial translocation to the portal vain, lymph nodes, systemic circulation, and thoracic duct very early after liver reperfusion. In their model, gut injury can only be attributed to remote ischemia and reperfusion from the liver. Portal congestion was eliminated, while the extent of hepatectomy was too limited to cause liver failure, and barrier failure was evident very early during reperfusion [67]. Filos et al. reported immediate and delayed gut barrier failure increasing bacterial translocation and endotoxaemia after 30 minutes of liver ischemia in rats. In addition, they found increased apoptosis in the intestinal mucosa 30 min after reperfusion [88]. Bedurlu et al. reported increased levels of TNF-*α*, bacterial translocation, and endotoxaemia 48 hours after hepatectomy performed under liver ischemia and reperfusion [109]. Jiang et al. showed that remote injury of the intestinal mucosa takes place after 30 minutes of liver ischemia followed by reperfusion in a rat experimental model. Microscopic study revealed denuded villi, disintegration of the lamina propria, appearance of exposed capillaries, and infiltration of neutrophils and macrophages in the ileal mucosa. In addition, enterocyte apoptosis was found to be increased after liver ischemia and reperfusion. Splenectomy reversed these findings suggesting a role of the spleen in inflammatory and apoptotic pathways, through neutrophil activation and cytokine expression [110]. Leister et al. concluded that partial hepatic ischemia/reperfusion injury leads to significant alterations of small bowel microcirculation and mucosal injury, while vasoactive intestinal polypeptide and gastrin-releasing peptide attenuated the damage [111]. Ochiai et al. showed increased intestinal permeability, endotoxinemia, and morphologic changes in the intestinal mucosa after liver ischemia and reperfusion combined with hepatectomy. These findings were reversed in the group treated with I2 prostaglandin analogues [112]. Finally, Meyer et al. have shown that liver ischemia and reperfusion increases the expression of ICAM in various organs including the intestine, implicating a possible mechanism of multiple organ failure [32].

Most of the experimental studies have been done in small animal models and, as a result, vascular occlusion is accompanied by transient portal hypertension. This is a major confounding factor, as direct damage to the intestinal mucosa can take place during splanchnic congestion. Only when this factor has been eliminated can the injury be attributed to remote injury by spillage of toxic substances from the liver.

Our team has developed an experimental model of liver ischemia and reperfusion injury combined with major hepatectomy, as a clinical analogue to major hepatectomy under vascular control [2]. In this model we have demonstrated that liver ischemia and reperfusion produces reactive oxygen species that result in oxidative injury to the intestinal mucosa. Intestinal mucosa oxidative markers were increased, suggesting a causative association with intestinal mucosa injury, apoptosis of enterocytes, and bacterial and endotoxin translocation. These parameters were reversed when antioxidant therapy was administered [6].

Clinical data regarding remote intestinal injury after liver ischemia/reperfusion derive mostly from liver transplantation series, because in liver resection studies addressing gut dysfunction, vascular control is not always used and, as a result, liver ischemia/reperfusion injury is not always present. In a randomized clinical study from Abdala et al. increased endotoxin translocation from the gut was reported following liver transplantation. The patients were divided in two groups, undergoing liver transplantation with a venovenous shunt or with the “piggyback” method. No endotoxin clearance was documented through the grafts of either group 2 hours after reperfusion [87]. In another study from Ronholm et al. postreperfusion hypotension observed after reperfusion of the graft was attributed to complement cascade activation originating from the gut during the reperfusion phase of orthotopic liver transplantation [113]. Wu et al. also demonstrated bacterial translocation in a clinical study of cirrhotic patients undergoing liver transplantation [114]. However, in clinical studies, no direct measurements of intestinal tissue injury have been made and there is no direct proof if mucosal injury exists, if it is the result of liver vascular manipulations, or if it preexisted prior to transplantation as a result of liver parenchyma injury.

**Figure 3:**
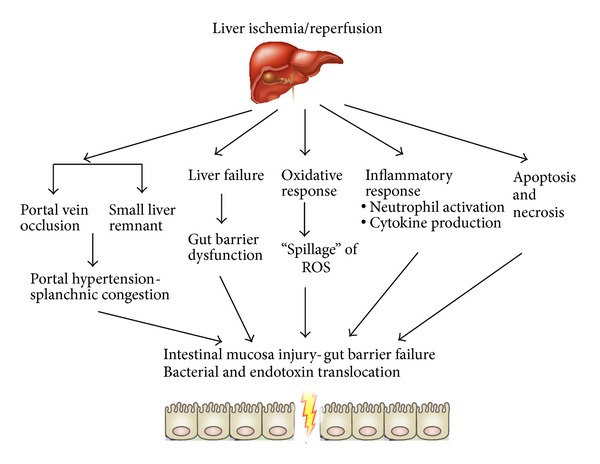
Mechanisms involved in remote injury to the gut mucosa leading to gut barrier failure after liver ischemia/reperfusion.

The above mechanisms are summarized in Figure 3.

## 6. Pancreatic Injury

Pancreatic dysfunction has been reported following major liver resections both in clinical trials [115] and in experimental studies [116]. The etiology of pancreatic dysfunction after these operations has not yet been clarified. However, among the risk factors proposed to interpret hyperamylasemia following hepatectomy are chronic liver disease and extent of hepatectomy, as well as portal congestion caused by the vascular control of the liver during these surgical procedures [117]. In a clinical study, Miyagawa et al. reported that the use of the Pringle maneuver during liver resection increases the incidence of postoperative hyperamylasemia and that the severity of hyperamylasemia is increased as the vascular occlusion time is prolonged [118]. Hashimoto et al. described the same conclusion in their clinical study reporting attenuated pancreatic injury in patients who were subjected to selective vascular control techniques [119]. Although the fist assumption of pancreatic injury after hepatectomy proposed portal congestion during vascular control as being responsible, Kubota et al. carried out a series of hepatectomies, occluding the superior mesenteric artery as well, during hepatic vascular control and thus eliminating any increase in portal pressure [120]. There were no differences in amylase compared with patients with intraoperative portal hypertension, implying that the possible mechanism was remote ischemia/reperfusion injury of the liver. Meyer et al. have reported ICAM-1 upregulation in remote organs, after liver ischemia and reperfusion in rats, including the pancreas, intestine, kidney, and lung [121]. The proposed mechanism is oxidative damage, derived from ROS production during liver ischemia/reperfusion by the use of hepatic vascular control during surgery. Ochiai et al. have also demonstrated pancreatic tissue injury after partial hepatectomy combined with ischemia/reperfusion in rats. They demonstrated a peak of acinar cell necrosis at 24 hours postoperatively as well as increased apoptotic activity. These findings were attenuated after treatment with I2 prostaglandin analogues [122]. Yang et al. found increased pancreatic MDA content, decreased pancreatic SOD activity, and increased serum amylase after 6 hours of reperfusion in a rodent model of 45-minute hepatic ischemia and reperfusion [123].

Our team has studied the pathophysiology of pancreatic injury following hepatic ischemia/reperfusion combined with hepatectomy. The key factor of posthepatectomy pancreatitis is thought to be the production of ROS, resulting in remote organ injury. In our study we measured the MDA content of portal blood and pancreatic tissue samples as a marker of lipid peroxidation and oxidative damage. Portal blood and pancreatic tissue MDA content was increased during liver reperfusion. In addition, we found increased amylase and c-peptide levels during reperfusion and histological evidence of pancreatic necrosis [124]. These data support the hypothesis that ROS and oxidative stress, as assessed by lipid peroxidation and tissue necrosis, play a crucial role in pancreatitis following liver ischemia/reperfusion combined with hepatectomy. Further evidence from our team supports the aforementioned hypothesis. Antioxidant administration (desferrioxamine) attenuated pancreatic injury after major hepatectomy under vascular control in a porcine experimental model, possibly by preventing and reversing production and circulation of oxidative products [54].

Pancreatic injury, in the form of acute pancreatitis, is a rare but severe complication of liver transplantation in children and adults [125]. Although multiple factors have been implicated, it seems that remote oxidative burst plays a pivotal role. In a recent study in rodents from Li et al. pancreatic injury following liver transplantation was found to be oxidative stress dependant. They found increased MDA content in pancreatic tissue, increased amylase and lipase serum levels, and morphological changes. These were attenuated with antioxidant treatment [126].

## 7. Adrenal Injury

Adrenal function during liver ischemia/reperfusion has not been adequately studied to date. However, there are reports of macroscopic injury to the adrenals following hepatectomy under vascular control and orthotopic liver transplantation [127, 128]. In addition, relative adrenal insufficiency has been reported in patients undergoing liver transplantation, even in steroid-free immunosuppressant protocols, in an incidence of up to 92% [129]. This has been described as the “hepatoadrenal” syndrome. A possible proposed mechanism is the decreased levels of high density lipoprotein (HDL) after liver transplantation and thus decreased cortisol synthesis. HDL levels have been shown to be a predictor of posttransplant relative adrenal insufficiency [130]. During the anhepatic phase, the liver does not produce apoA-1 (which is essential for the formation of HDL) for a number of hours. In addition, the transplanted liver suffers ischemia/reperfusion injury that is responsible for postoperative liver dysfunction. ApoA-1 has a relatively short half-life and, therefore, the HDL levels are decreased after transplantation.

Liver ischemia/reperfusion has been shown to increase the levels of endotoxin, as well as various cytokines, including TNF-*α*, IL-1*β*, and IL-6. Endotoxin and TNF-*α* have been shown to inhibit steroidogenesis. Endotoxin has been reported to bind to the HDL receptor, neutralizing it [131]. Increased levels of TNF-*α*, IL-1*β*, and IL-6, which are produced during hepatic ischemia/reperfusion, have been shown to decrease synthesis and secretion of apoA-1 [132]. In addition, TNF-*α* has been demonstrated to directly inhibit steroidogenesis and increase resistance to cortisol [130].

## 8. Myocardial Injury

Although indications about a connection between acute liver injury and myocardial damage had already been reported [133, 134], this was brought sharply into focus in a study by the US Acute Liver Failure Study Group which showed that elevation of serum troponin I (cTnI) levels is common (74% overall) in patients with ALF of various etiologies [135]. However, in both this and the previous studies, liver ischemia/reperfusion was absent as a mechanism of injury. Furthermore, these studies focused more on clinical aspects and less on pathophysiology. Tanaka et al. showed that liver ischemia/reperfusion is accompanied by myocardial cell necrosis (manifested by elevated troponin levels) and proposed ROS production as a putative mechanism of injury [25]. However, this study suffered from a major design limitation: to achieve liver ischemia/reperfusion, the portal vein was totally occluded, leading to intestinal congestion during liver ischemia, with unknown effects in the observed results. A similar follow-up study also documented myocardium injury after total hepatic ischemia and reperfusion and reversal with antioxidant treatment. However, this study had similar limitations, as the inferior vena cava was also occluded in the process, leading to certain hemodynamic instability and altered cardiac output, making pathophysiologic mechanisms unclear [54]. Recently, the same group published data showing attenuation of myocardium injury after total hepatic ischemia and reperfusion after the administration of tacrolimus, implicating inflammatory mechanisms in the pathophysiology as well [5].

Trying to overcome these limitations, our team designed a porcine model of liver ischemia/reperfusion that diverted portal blood flow during ischemia to the inferior vena cava and restored normal flow path during reperfusion. Our results showed a relatively subtle yet consistent injury to the myocardium early after liver ischemia/reperfusion, manifested mainly by increase in cardiac troponin I blood levels and confirmed histologically by myocardiocyte necrosis [136].

An unexpected result of this study was the early myocardial injury (prior to 6 hours after reperfusion), certainly before acute liver failure manifestations (e.g., increase in liver enzymes, intracranial hypertension, and hemodynamic instability). It is therefore speculated that myocardial injury was probably attributed to liver ischemia/reperfusion and not to the ensuing acute liver failure. A putative mechanism involves the generation of reactive oxygen and nitrogen species during liver ischemia/reperfusion. However, myocardial damage in this setting was subclinical and mostly evident under microscopic examination; therefore the heart might be one of the least clinically affected organs in liver ischemia/reperfusion. It must not be forgotten, however, that all experimental as well as most clinical observations are made in relatively healthy hearts and not those burdened with ischemia, cardiomyopathy, and/or systolic or diastolic dysfunction.

## 9. Comments

Liver ischemia/reperfusion injury has been shown to play a pivotal role in the pathogenesis of the frequently observed remote organ injury following a variety of clinical scenarios including hepatic surgery and transplantation. Multiple mechanisms have been implicated in the pathophysiology of this remote organ injury. Among them, the oxidative and the inflammatory pathway have been shown to play an important role. However, most of the available studies of remote organ injury after hepatic ischemia/reperfusion examine the liver and the respective organ* in vivo*. Therefore the employed therapeutic strategies could be hypothesized to ameliorate remote organ injury through attenuation of liver injury, without knowing their net effect on remote organ injury alone. A possible exception could be the experimental models of isolated perfused organs. Although the former strategy is clearly more applicable to the clinical scenario, the latter methodology could be more helpful in the elucidation and clarification of the underlying mechanisms implicated in the pathophysiology of hepatic ischemia/reperfusion-induced remote organ injury. One of course could argue that remote organ injury is dependent on cellular and molecular events following liver reperfusion and could be effectively attenuated with appropriate interventions to reduce hepatic ischemia/reperfusion injury. Such interventions can be targeted towards specific molecular mechanisms, as mentioned above, or towards more holistic methods such ischemic preconditioning or bioartificial liver devices. Finally, since most of the current knowledge on remote organ injury derives from experimental studies, the investigation of this entity and the assessment of the efficacy of the various therapeutic strategies in the clinical setting are warranted.
